# Pre-Loss Group Therapy for Family Caregivers of Persons With Dementia: Translation Into Practice

**DOI:** 10.1093/geroni/igaa057.139

**Published:** 2020-12-16

**Authors:** Katherine Supiano, Troy Andersen, Marilyn Luptak, Cynthia Beynon, Eli Iacob, Hui Li

**Affiliations:** University of Utah, Salt Lake City, Utah, United States

## Abstract

We developed Pre-Loss Group Therapy (PLGT) for dementia caregivers at risk for Complicated Grief (CG). PLGT is a manualized ten-session multi-modal group therapy that includes elements of cognitive behavior therapy, motivational interviewing, exposure therapy, memory revisiting, meaning-making, and self-care. We implemented and evaluated three PLGT cohorts in three long-term care facilities with family caregivers at-risk for CG whose care recipient had a life expectancy of 6 months or less and resided in a long-term care facility (NT = 24). Evaluation of participant preparedness for the death of the persons with dementia (PWD), self-care and grief outcomes showed significant improvement across multiple domains between pre and post-group, notably a statistically significant decrease in grief as measured by the Inventory of Complicated Grief score from baseline (M = 25.67, SE=1.80) to post-group (M = 14.41, SE=1.65) t(21)= 6.280, p<0.001. Clinician-rated grief severity declined (N=22, β = –0.472, SE = 0.018, p < 0.001) per week and grief improvement increased (N=22, β = 0.259, SE = 0.023, p < 0.001) per week, as assessed on the Clinician Global Impressions Scale. We subsequently trained two LCSWs to conduct PLGT, and both clinical outcomes and treatment fidelity and skills measures achieved performance levels of master clinician-trainers. Family caregivers at risk for CG may benefit from group therapy targeting preparedness and pre-loss grief experience, as we provide with PLGT. Manualized PLGT is suitable for implementation by LCSWs in the settings of hospice and long-term care.

